# Metformin-Induced MicroRNA-34a-3p Downregulation Alleviates Senescence in Human Dental Pulp Stem Cells by Targeting CAB39 through the AMPK/mTOR Signaling Pathway

**DOI:** 10.1155/2021/6616240

**Published:** 2021-01-06

**Authors:** Shuo Zhang, Rong Zhang, Pengyan Qiao, Xiaocao Ma, Rongjian Lu, Feifan Wang, Chuanjie Li, Lingling E, Hongchen Liu

**Affiliations:** ^1^Medical College of Nankai University, Tianjin 300071, China; ^2^Institute of Stomatology & Oral Maxilla Facial Key Laboratory, Chinese PLA General Hospital, Beijing 100853, China; ^3^Department of Stomatology, PLA Strategic Support Force Characteristic Medical Center, Beijing 100101, China; ^4^Department of Stomatology, Fifth Medical Center of Chinese PLA General Hospital, Beijing 100071, China; ^5^Department of Temporomandibular Joint Surgery, Affiliated Stomatology Hospital of Guangzhou Medical University, Guangzhou 510182, China

## Abstract

Dental pulp stem cells (DPSCs) are ideal seed cells for the regeneration of dental tissues. However, DPSC senescence restricts its clinical applications. Metformin (Met), a common prescription drug for type 2 diabetes, is thought to influence the aging process. This study is aimed at determining the effects of metformin on DPSC senescence. Young and aging DPSCs were isolated from freshly extracted human teeth. Flow cytometry confirmed that DPSCs expressed characteristic surface antigen markers of mesenchymal stem cells (MSCs). Cell Counting Kit-8 (CCK-8) assay showed that a concentration of 100 *μ*M metformin produced the highest increase in the proliferation of DPSCs. Metformin inhibited senescence in DPSCs as evidenced by senescence-associated *β*-galactosidase (SA-*β*-gal) staining and the expression levels of senescence-associated proteins. Additionally, metformin significantly suppressed microRNA-34a-3p (miR-34a-3p) expression, elevated calcium-binding protein 39 (CAB39) expression, and activated the AMP-activated protein kinase (AMPK)/mammalian target of rapamycin (mTOR) signaling pathway. Dual-luciferase reporter assay confirmed that CAB39 is a direct target for miR-34a-3p. Furthermore, transfection of miR-34a-3p mimics promoted the senescence of DPSCs, while metformin treatment or Lenti-CAB39 transfection inhibited cellular senescence. In conclusion, these results indicated that metformin could alleviate the senescence of DPSCs by downregulating miR-34a-3p and upregulating CAB39 through the AMPK/mTOR signaling pathway. This study elucidates on the inhibitory effect of metformin on DPSC senescence and its potential as a therapeutic target for senescence treatment.

## 1. Introduction

Stem cells are used for restoring injured tissues because of their self-renewal, multipotent differentiation, and paracrine signaling properties. Stem cells derived from dental tissues, such as dental pulp, tooth germ, apical papilla, and periodontal ligament, are abundant in number and easily accessible. They are considered attractive candidates for tissue engineering and regenerative medicine [1]. Dental pulp stem cells (DPSCs) were the first human dental mesenchymal stem cells (MSCs) to be isolated from pulp tissue [2]. These cells can undergo chondrogenic, osteogenic, adipogenic, neurogenic, and odontogenic differentiation [3] and can, therefore, be used for dental tissue regenerative therapy in pulp revascularization, dentine formation, and periodontal regeneration [4].

Senescence is considered an inescapable and irreversible state, which is characterized by specific changes in cell morphology, function, and gene expression [5] and causes the increased occurrence of age-related diseases. With increasing age, the number, regenerative capacity, biological activity, and resistance to oxidative stress of stem cells decrease [6–8]. Studies have reported that senescence suppresses the proliferative capacity and differentiation potential of DPSCs [9]. For instance, senescence affects the ability of DPSCs to influence the mineralization processes and decreases the osteogenic potential of aged human DPSCs [10]. This suggests that DPSC senescence is likely to exert negative adverse effects on their clinical applications in cell-based therapies. Therefore, an in-depth understanding of the molecular mechanisms of DPSC senescence and their potential as targets for drug design is an important research direction for senescence inhibition.

Metformin (Met), a first-line prescription drug for type 2 diabetes mellitus, has been proven to influence the aging process [11]. Preclinical studies revealed that metformin exhibited prolongevity and health span-extending properties in *Caenorhabditis elegans*, mice, and human cell lines [12–15]. Metformin also reduces the incidence of human age-related diseases [16] and has, thus, been proposed as a drug candidate for slowing aging in humans [17]. Mechanistically, metformin-mediated gero-suppressive effects on metabolism mainly depend on the activation of AMP-activated protein kinase (AMPK), which leads to the downstream inhibition of the mammalian target of rapamycin (mTOR) [18]. AMPK/mTOR signaling pathway has been widely used and well-documented in current studies to counteract senescence and is considered a crucial intracellular mechanism of metformin in attenuating the hallmarks of senescence [19]. Furthermore, the calcium-binding protein 39 (CAB39), a scaffold protein of liver kinase B1 (LKB1), activates the phosphorylation of AMPK and is, therefore, an upstream kinase of the AMPK/mTOR signaling pathway [20]. However, it has not been established whether metformin mediates DPSC senescence by regulating CAB39/AMPK/mTOR signaling. Moreover, the potential underlying mechanisms have not been elucidated.

MicroRNAs (miRNAs), a type of endogenous small-molecule noncoding RNA, have been reported to play crucial roles in mediating cellular senescence. The microRNA-34a (miR-34a) has been regarded as a potential sensor of senescence, since its expression is increased in several aged tissues and cells, including DPSCs [21–24]. Additionally, metformin has a regulatory effect on miR-34a, where it regulates multiple biological processes by up- or downregulating the expression of miR-34a [25–28]. A previous study demonstrated that miR-34a exhibited an antiangiogenic action in mouse microvascular endothelial cells, which could be modulated by metformin [25]. In another study, metformin was found to attenuate high glucose-stimulated fibrosis and inflammation in rat mesangial cells by negatively regulating miR-34a [26]. Numerous studies have revealed that miR-34a is a direct target of metformin. However, deeper insights between metformin and miR-34a are still lacking in DPSC senescence.

This study is aimed at determining the effects of metformin on miR-34a-3p and investigating the molecular mechanisms underlying the function of miR-34a-3p, specifically focusing on its target gene CAB39 and the AMPK/mTOR signaling pathway, in regulating the senescence of DPSCs.

## 2. Materials and Methods

### 2.1. Pulp Samples

Human dental pulp tissues were harvested from 12 healthy donors undergoing tooth extraction for therapeutic or orthodontic reasons without pulp inflammation, severe periodontitis, or systemic disease. The samples were classified into two age groups (6 donors per group): the young group (18–27 years) and the aging group (65–74 years). All patients provided a written informed consent prior to participation, and the experimental protocol was approved by the Medical Ethics Committee of Chinese People's Liberation Army (PLA) General Hospital (ethics approval no. S2018-094-01).

### 2.2. Cell Culture

The primary culture of DPSCs was carried out by an enzyme digestion method. Briefly, the dental pulp tissues were obtained from the crown and superior two thirds of the root pulp. They were then cut into small fragments and digested for 1 h at 37°C in a solution of 3 mg/ml type I collagenase (Worthington Biochemical) and 4 mg/ml dispase (Sigma). Primary DPSCs were maintained in complete *α*-MEM medium supplemented with 20% fetal bovine serum (Invitrogen) and 100 U/ml penicillin/streptomycin (Invitrogen). The culture was incubated in a humidified 5% CO_2_ incubator at 37°C. The media was changed every 3 days, and cell passage was performed when the cells reached 80–90% confluence. The growth status of young and aging DPSCs was monitored and recorded under an optical microscope (×100 magnification; Olympus, Japan). DPSCs at the fourth passage were used to carry out a series of experiments.

### 2.3. Flow Cytometry Analysis

Flow cytometry was performed to detect human DPSCs surface markers. Young and aging DPSCs were collected, washed with phosphate-buffered saline (PBS), and fixed in 4% paraformaldehyde (PFA). After fixation for 30 min, the cells were permeabilized and incubated with antibodies for 1 h at 4°C. The following antibodies specific for human surface antigens were used: CD44-FITC (Biolegend, 103005), CD90-FITC (Biolegend, 328107), CD105-PE (eBioscience, 4300023), CD11b-FITC (eBioscience, 4271325), CD14-FITC (Biolegend, 301803), CD34-PE (Biolegend, 119307), and CD45-FITC (Biolegend, 304005). The corresponding isotype-matched (IgG) antibodies conjugated to PE and FITC served as negative controls. Data were analyzed using the FlowJo software (FlowJo, Ashland, OR, USA).

### 2.4. Cell Proliferation: Identification of Metformin Dose

To determine the concentration of metformin to be used in subsequent experiments, the proliferation capacity of young and aging DPSCs treated with various concentrations of metformin (10, 50, 100, 250, and 500 *μ*M) was determined by Cell Counting Kit-8 (CCK-8) assay (Dojindo, Japan). Samples without added metformin served as controls. Cells were inoculated in 96-well culture plates at a density of 2 × 10^3^ cells/well for 24 h and refreshed with a maintenance medium containing metformin at different concentrations for 1 to 10 days (*n* = 8). The medium was removed, and the cells were washed with PBS. To each well, 110 *μ*l solution (100 *μ*l *α*-MEM and 10 *μ*l CCK-8) was added, and the plate was incubated for 1 h at 37°C. Absorbance was then measured at 450 nm wavelength with an enzyme-labeling instrument (Tecan Infinite 200 Pro, Switzerland).

### 2.5. Senescence-Associated *β*-Galactosidase (SA-*β*-gal) Staining

SA-*β*-gal staining was conducted using a SA-*β*-gal staining kit (Solarbio, Beijing, China) to detect the senescence of young and aging DPSCs. Briefly, cells were fixed in 4% PFA for 15 min at room temperature, washed three times with PBS, and incubated with SA-*β*-gal staining solution at 37°C overnight. The complete *β*-gal staining solution contained 40 mM sodium phosphate (pH 6.0), 5 mM potassium ferrocyanide, 5 mM potassium ferricyanide, 150 mM NaCl, 2 mM MgCl_2_, and 1 mg/ml X-Gal. The sections were washed with PBS, mounted in glycerol, and visualized under an Olympus IX71 microscope (Olympus, Japan). SA-*β*-gal-positive cells appearing as blue-stained cells were randomly imaged. The percentage of aging DPSCs was determined as the ratio of positive DPSCs to the total number of DPSCs obtained from five different fields of view.

### 2.6. Transfection of miR-34a-3p Mimics

The miR-34a-3p mimics and the corresponding negative control (NC) were chemically synthesized by Integrated Biotech Solutions (Shanghai, China). The miR-34a-3p mimics and miR-NC sequences were 5′-CAAUCAGCAAGUAUACUGCCCU-3′ and 5′-UCACAACCUCCUAGAAAGAGUAGA-3′, respectively. Lipofectamine 2000 transfection reagent (Invitrogen) was used to perform transient transfection of the oligonucleotides according to the manufacturer's instructions. Aging DPSCs were pretransfected with miR-34a-3p mimics or miR-NC to a final incubation concentration of 100 nM for 48 h, before stimulation with metformin for 48 h. The transfection efficiency was examined by reverse transcription-quantitative polymerase chain reaction (RT-qPCR).

### 2.7. Dual-Luciferase Reporter Assay

The fragment of the wild-type (WT) 3′UTR of CAB39 predicted to interact with miR-34a-3p and mutant (MUT) CAB39-3′UTR (GenePharma, Shanghai, China) was amplified and subcloned into the pmirGLO vector (Promega Corporation, Madison, WI, USA). The constructed luciferase reporter plasmids were named CAB39-3′UTR WT and CAB39-3′UTR MUT, respectively. For dual-luciferase reporter assay, HEK-293T cells were used because of their high transfection efficiency [29]. Briefly, HEK-293T cells were seeded into 24-well plates before transfection. When the cells reached 70 to 80% confluency, cotransfection with either miR-34a-3p mimics or miR-NC and either WT or MUT reporter plasmids was performed using Lipofectamine 2000. After transfection for 48 h at 37°C, the cells were collected, and luciferase activity was analyzed using a Dual-Luciferase Reporter Assay System (Promega Corporation, Madison, WI, USA).

### 2.8. Lentivirus Construction and Transfection

To overexpress the CAB39 gene, lentiviral plasmids were purchased from SyngenTech (Shanghai, China). For lentivirus constructs, the coding sequence of CAB39 was inserted into the pHS-AVC-LY027 lentiviral vector. The primer sequences used were as follows: forward primer, 5′-TAAGATCTACAGCTGCCTTG-3′, and reverse primer, 5′-TGACATTTCGACATATCTGA-3′. Lentiviral stocks were produced in HEK-293FT cells (SyngenTech, Shanghai, China) according to the manufacturer's instructions. Briefly, the virus-containing medium was collected 48 h after transfection and filtered through a 0.45 *μ*m filter. To establish stable cell lines, lentiviral transfection was performed by replacing the medium with a virus-containing medium in the presence of 8 *μ*g/ml polybrene. At 48 h posttransfection, the transfection efficiency was measured by RT-qPCR.

### 2.9. RT-qPCR

The expression levels of miR-34a-3p and CAB39 were determined by RT-qPCR. Total RNA was isolated from DPSCs using the TRIzol reagent (Invitrogen, Beijing, China) and reverse transcribed to generate cDNA using a PrimeScript RT Reagent Kit (TIANGEN Biotech, Beijing, China) according to the manufacturer's instructions. The RT-qPCR reactions were performed using SYBR-Green PCR Master Mix (TransGen Biotech, Beijing, China) with the ABI7500 System (Applied Biosystems; Thermo Fisher Scientific, Inc., Waltham, MA, USA). The expression of U6 snRNA and *β*-Actin served as internal controls to normalize the miRNA and mRNA expression levels, respectively. Relative expression levels of miR-34a-3p and CAB39 were calculated using the 2^-*ΔΔ*CT^ cycle threshold method. The primer sequences used were as follows: miR-34a-3p forward sequence, 5′-CAATCAGCAAGTATACTGCCT-3′, U6 forward sequence, 5′-CGCAAGGATGACACGCAAATTC-3′, and the universal reverse primer sequence of miR-34a-3p and U6, 5′-GTGCAGGGTCCGAGGT-3′; CAB39 forward sequence, 5′-AAATCTCCAGCAGACATTGTG-3′, and reverse sequence, 5′-CAAGTCAATGAGCTGTAAATCA-3′; and *β*-Actin forward sequence, 5′-CATGTACGTTGCTATCCAGGC-3′, and reverse sequence, 5′-CTCCTTAATGTCACGCACGAT-3′.

### 2.10. Western Blotting

The protein levels of CAB39, phosphorylated (p-) AMPK, AMPK, p-mTOR, mTOR, p53, p21, and p16 were determined by western blotting. Briefly, total proteins of DPSCs were extracted using RIPA buffer (Beyotime, Shanghai, China). Protein concentrations were measured using the bicinchoninic acid (BCA) protein assay kit (Beyotime, Shanghai, China). A total of 30 *μ*g equal protein samples were resolved in 10% sodium dodecyl-sulfate-polyacrylamide gel electrophoresis (SDS-PAGE) and transferred onto polyvinylidene fluoride (PVDF) membranes (Millipore, Darmstadt, Germany). 5% nonfat milk was used to block the membrane in TBST for 2 h at room temperature. The membranes were incubated at 4°C overnight with the following primary antibodies: anti-CAB39 (Abcam, ab51132), anti-p-AMPK (CST, 2535S), anti-AMPK (Proteintech, 66536-1-Ig), anti-p-mTOR (Abcam, ab109268), anti-mTOR (Abcam, ab32028), anti-p53 (Proteintech, 10442-1-AP), anti-p21 (Proteintech, 10355-1-AP), anti-p16 (Proteintech, 10883-1-AP), and anti-*β*-Actin (Proteintech, 20536-1-AP). Subsequently, the membranes were washed three times with TBST and incubated with secondary antibodies (1 : 1000, Beyotime, Shanghai, China) for 1 h at 37°C. *β*-Actin was used as an internal control. All protein blots were chemiluminated using an enhanced chemiluminescence (ECL) detection kit (High-sig ECL Western Blotting Substrate; Tanon, Shanghai, China).

### 2.11. Statistical Analysis

All experiments were repeated at least three independent times. Data were analyzed using GraphPad Prism 5.0 statistical program (GraphPad Software, La Jolla, CA, USA) and presented as the mean ± standard deviation (SD). Comparisons between two groups were assessed by Student's *t*-test, whereas multigroup comparisons were analyzed by one-way ANOVA followed by Tukey's multiple comparison test. A *p* value of < 0.05 was considered statistically significant.

## 3. Results

### 3.1. Isolation, Culture, and Identification of DPSCs

**Figure 1:**
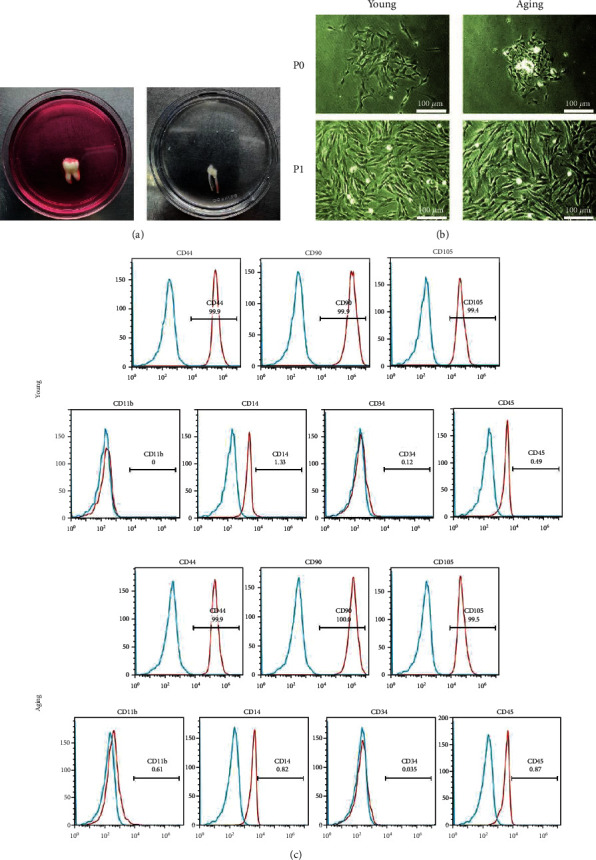
Isolation, culture, and identification of DPSCs. (a) The human pulp tissues were obtained from freshly extracted teeth. (b) Young and aging DPSCs were observed at P0 and P1 using an optical microscope (magnification, ×100). (c) Surface marker profiling of young and aging DPSCs was evaluated by flow cytometry.

The human pulp tissues were isolated from freshly extracted teeth of young and aging donors (Figure 1(a)). Primary DPSCs were cultured using an enzymatic digestion method. The morphology of wall-adherent cells was long spindle-shaped or polygonal under the inverted optical microscope (Figure 1(b)). Flow cytometry demonstrated that both young and aging DPSCs positively expressed human MSC surface markers CD44 (99.9%, 99.9%), CD90 (99.9%, 100%), and CD105 (99.4%, 99.5%), while negatively expressing hematopoietic cell markers CD11b (0%, 0.61%), CD14 (1.33%, 0.82%), CD34 (0.12%, 0.035%), and CD45 (0.49%, 0.87%) (Figure 1(c)). These results indicated that DPSCs conformed to the characteristics of MSCs.

### 3.2. The Influence of Metformin Treatment on DPSC Proliferation

**Figure 2:**
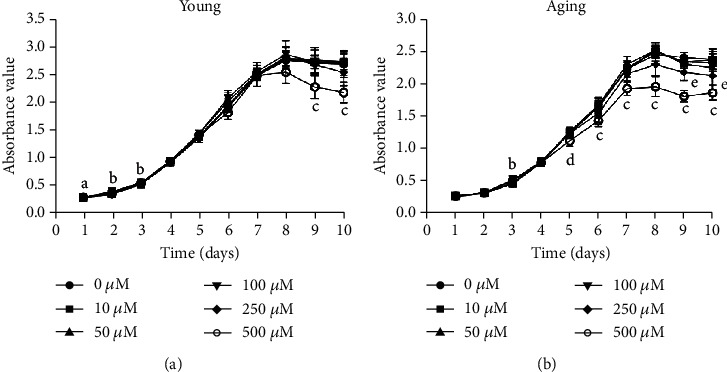
The influence of metformin treatment on DPSC proliferation. (a) The influence of metformin on young and (b) aging DPSC proliferation. Young and aging DPSCs were treated with 10, 50, 100, 250, and 500 *μ*M metformin for 1 to 10 days; cells with no drug added (0 *μ*M) served as controls. Cell proliferation was determined by CCK-8 assay. The absorbance value was read at a wavelength of 450 nm. a, *p* < 0.01, 100 *μ*M versus 0 *μ*M; b, *p* < 0.05, 100 *μ*M versus 0 *μ*M; c, *p* < 0.01, 500 *μ*M versus 0 *μ*M; d, *p* < 0.05, 500 *μ*M versus 0 *μ*M; e, *p* < 0.01, 250 *μ*M versus 0 *μ*M.

The influence of metformin on cell proliferation was evaluated, and a suitable therapeutic concentration was established. The proliferation capacity assessed by CCK-8 showed a nonlinear metformin dose-dependent curve. Compared to the control group, 100 *μ*M metformin significantly promoted the proliferation of young DPSCs on the first three days and of aging DPSCs on the third day (*p* < 0.05, Figures 2(a) and 2(b)). However, 250 *μ*M metformin significantly inhibited the proliferation of aging DPSCs on the ninth and tenth days (*p* < 0.01, Figure 2(b)). Furthermore, metformin at a high concentration (500 *μ*M) decreased the proliferation activity of young DPSCs on the ninth and tenth days and of aging DPSCs after the fifth day (*p* < 0.05, Figures 2(a) and 2(b)). Based on these findings, 100 *μ*M metformin was selected as the concentration to be used in subsequent experiments.

### 3.3. Metformin Inhibits Senescence in DPSCs

**Figure 3:**
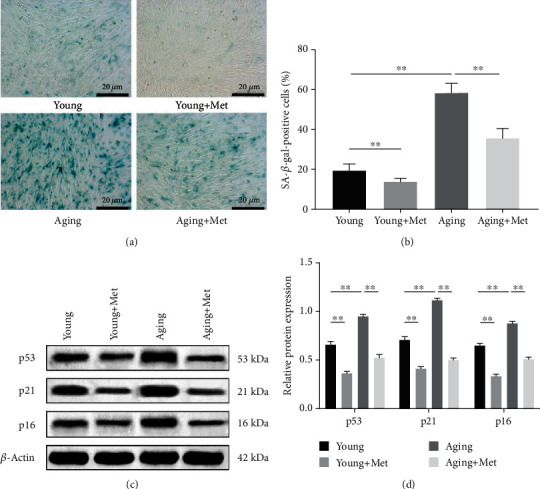
Metformin inhibits senescence in DPSCs. Young and aging DPSCs were treated with 100 *μ*M metformin for 48 h. (a) Cellular senescence was determined using SA-*β*-gal staining. Representative images are shown (scale bar: 20 *μ*m). (b) Quantitative analysis of SA-*β*-gal-positive cells. (c) Representative blots of p53, p21, and p16 protein expression levels by western blotting. (d) Quantitative analysis of p53, p21, and p16 proteins. Data are expressed as the mean ± standard deviation (*n* = 3). ^∗∗^*p* < 0.01, with comparisons indicated by lines.

The SA-*β*-gal staining was performed to identify the effect of metformin on the senescence of young and aging DPSCs. The results showed that the percentage of SA-*β*-gal-positive cells in aging DPSCs was markedly higher than that in young DPSCs (*p* < 0.01, Figures 3(a) and 3(b)). However, when treated with metformin, the percentage of SA-*β*-gal-positive cells significantly decreased in both young and aging groups (*p* < 0.01, Figures 3(a) and 3(b)), indicating that metformin could inhibit senescence in DPSCs.

The protein levels of p53, p21, and p16 were assessed by western blotting. The results demonstrated that metformin stimulation suppressed the levels of p53, p21, and p16 in young and aging DPSCs (*p* < 0.01, Figures 3(c) and 3(d)), suggesting that metformin inhibited the senescence of DPSCs by inhibiting the expression of senescence-associated proteins.

### 3.4. Metformin Inhibits miR-34a-3p Expression and Activates the AMPK/mTOR Signaling Pathway in DPSCs

**Figure 4:**
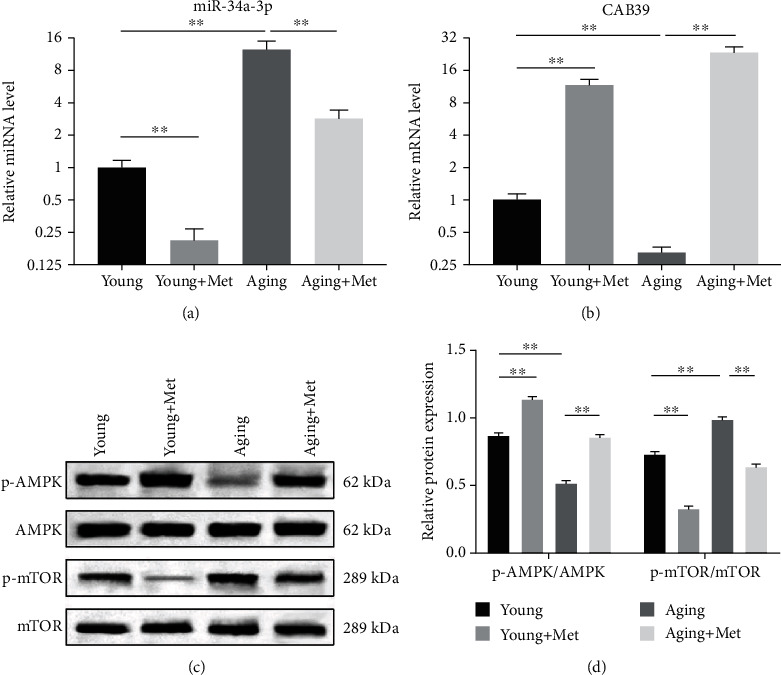
Metformin inhibits miR-34a-3p expression and activates the AMPK/mTOR signaling pathway in DPSCs. Young and aging DPSCs were treated with 100 *μ*M metformin for 48 h. (a) Relative expression levels of miR-34a-3p and (b) CAB39 were determined by RT-qPCR. (c) Representative blots of p-AMPK, AMPK, p-mTOR, and mTOR protein expression levels. (d) Quantitative analysis of the proportion of p-AMPK/AMPK and p-mTOR/mTOR. Data are expressed as the mean ± standard deviation (*n* = 3). ^∗∗^*p* < 0.01, with comparisons indicated by lines.

To investigate the effect of metformin on the expression of miR-34a-3p and downstream signaling pathway molecules, the expression of miR-34a-3p and CAB39 was determined by RT-qPCR, while the expression of p-AMPK, AMPK, p-mTOR, and mTOR was determined by western blotting. RT-qPCR results showed that in aging DPSCs, miR-34a-3p was significantly upregulated while CAB39 was significantly downregulated when compared to young DPSCs (*p* < 0.01, Figures 4(a) and 4(b)), suggesting that both miR-34a-3p and CAB39 might be associated with DPSC senescence. The expression of miR-34a-3p was significantly decreased following metformin treatment (*p* < 0.01, Figures 4(a) and 4(b)), whereas the expression of CAB39 was significantly increased in both young and aging DPSCs (*p* < 0.01, Figures 4(a) and 4(b)). These results clearly illustrated that metformin inhibited miR-34a-3p expression and induced CAB39 expression.

Western blotting analysis showed that the expression of p-AMPK was significantly downregulated, while the expression of p-mTOR was significantly upregulated in aging DPSCs when compared to young DPSCs (*p* < 0.01, Figures 4(c) and 4(d)), indicating that the AMPK/mTOR signaling was associated to the senescence of DPSCs. Similarly, in metformin-treated cells, the p-AMPK expression level was significantly elevated, whereas the p-mTOR expression level was significantly suppressed (*p* < 0.01, Figures 4(c) and 4(d)), indicating that the administration of metformin led to the activation of the AMPK/mTOR signaling pathway.

### 3.5. miR-34a-3p Downregulates CAB39 Expression

**Figure 5:**
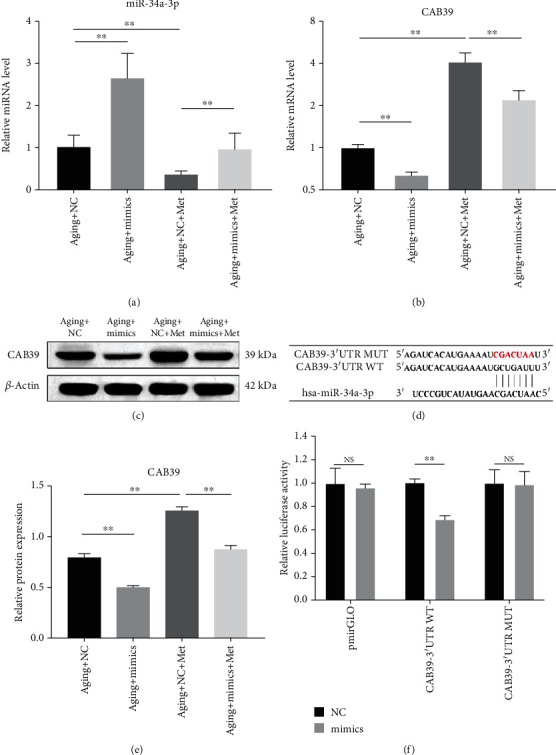
miR-34a-3p downregulates CAB39 expression. (a) Relative expression levels of miR-34a-3p and (b) CAB39 were determined by RT-qPCR in aging DPSCs transfected with miR-34a-3p mimics or miR-NC for 48 h followed by 100 *μ*M metformin for 48 h. (c) Representative blots and (e) quantitative analysis of CAB39 protein expression levels in aging DPSCs transfected with miR-34a-3p mimics or miR-NC for 48 h followed by 100 *μ*M metformin for 48 h. (d) The predicted potential binding site of miR-34a-3p at the 3′UTR of CAB39 in the human gene is shown. Meanwhile, a mutation was constructed where the letters in red indicate mutated nucleotides. (f) Relative luciferase activity. Data are expressed as mean ± standard deviation (*n* = 3). NS: not significantly different. ^∗∗^*p* < 0.01, with comparisons indicated by lines.

To verify the potential relationship between miR-34a-3p and CAB39, aging DPSCs were treated with metformin or miR-34a-3p mimics, and the expression levels of miR-34a-3p and CAB39 were determined. It was found that the expression level of miR-34a-3p was significantly increased following transfection with miR-34a-3p mimics and significantly decreased following metformin treatment (*p* < 0.01, Figure 5(a)), confirming the successful transfection of miR-34a-3p mimics. Additionally, the transfection of miR-34a-3p mimics led to a significant decrease in the CAB39 expression level, while the metformin-induced upregulation of CAB39 was attenuated at both the mRNA and protein levels (*p* < 0.01, Figures 5(b)–5(d)), indicating that miR-34a-3p downregulated the expression of CAB39.

To further examine the role of miR-34a-3p in the regulation of CAB39, a dual-luciferase reporter assay was performed. The potential miR-34a-3p binding site of CAB39-3′UTR was predicted by the TargetScan database (http://www.targetscan.org/), a bioinformatics prediction tool (Figure 5(e)). The relative luciferase activity was significantly downregulated when CAB39 WT was cotransfected with miR-34a-3p mimics when compared to miR-NC cotransfection (*p* < 0.01), while the inhibitory role was abolished when CAB39-3′UTR was mutated (*p* > 0.05, Figure 5(f)), indicating that miR-34a-3p was specifically bound to the predicted CAB39-3′UTR.

### 3.6. Metformin-Induced miR-34a-3p Downregulation Suppresses Senescence in DPSCs through the AMPK/mTOR Signaling Pathway

**Figure 6:**
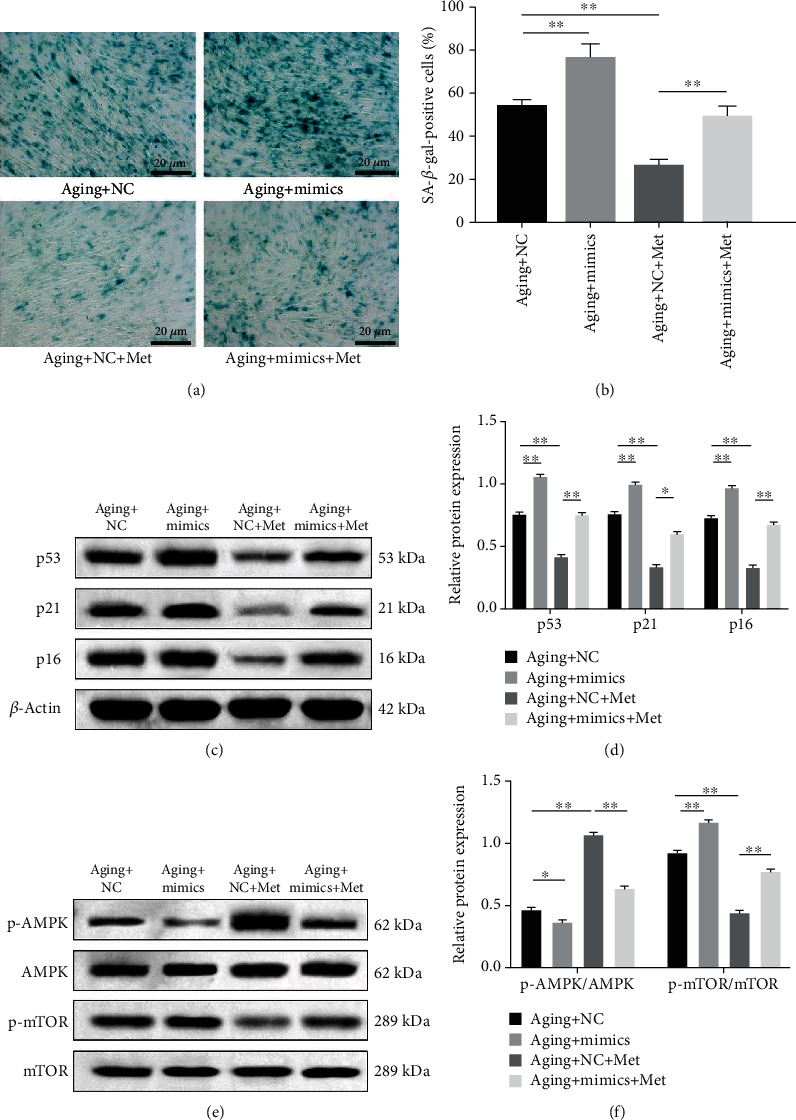
Metformin-induced miR-34a-3p downregulation suppresses senescence in DPSCs through the AMPK/mTOR signaling pathway. Aging DPSCs were transfected with miR-34a-3p mimics or miR-NC for 48 h and treated with 100 *μ*M metformin for 48 h. (a) SA-*β*-gal staining to determine senescence. Representative images are shown (scale bar: 20 *μ*m). (b) Quantitative analysis of SA-*β*-gal-positive cell rates. (c) Representative blots and (d) quantitative analysis of the expression levels of p53, p21, and p16 in aging DPSCs. (e) Representative blots of the expression levels of p-AMPK, AMPK, p-mTOR, and mTOR in aging DPSCs. (f) Quantitative analysis of p-AMPK/AMPK and p-mTOR/mTOR ratios. Data are expressed as mean ± standard deviation (*n* = 3). ^∗^*p* < 0.05, ^∗∗^*p* < 0.01, with comparisons indicated by lines.

To explore the role of miR-34a-3p in metformin-mediated inhibition of senescence, aging DPSCs were treated with metformin or miR-34a-3p mimics. The SA-*β*-gal staining results revealed that transfection of miR-34a-3p mimics significantly upregulated the ratio of SA-*β*-gal-positive cells (*p* < 0.01, Figures 6(a) and 6(b)). Additionally, the metformin inhibitory effect on the senescence of DPSCs was reversed by the overexpression of miR-34a-3p (*p* < 0.01, Figures 6(a) and 6(b)). Western blotting analysis showed that the transfection of miR-34a-3p mimics yielded a significant increase in the expression levels of senescence-associated proteins p53, p21, and p16 relative to those in the miR-NC group (*p* < 0.01, Figures 6(c) and 6(d)). Moreover, the metformin inhibitory effects on senescence-associated protein expression were attenuated upon miR-34a-3p overexpression in aging DPSCs (*p* < 0.05, Figures 6(c) and 6(d)). In addition, the expression of p-AMPK was reduced after transfecting miR-34a-3p mimics, while the expression of p-mTOR was elevated, indicating the activation of the AMPK/mTOR signaling pathway (*p* < 0.05, Figures 6(e) and 6(f)). It is also important to note that miR-34a-3p overexpression significantly abrogated metformin-mediated increase in p-AMPK/AMPK ratio and the decrease in p-mTOR/mTOR ratio (*p* < 0.01, Figures 6(e) and 6(f)). Collectively, this study demonstrated that miR-34a-3p aggravated DPSC senescence by activating the AMPK/mTOR signaling pathway, while metformin treatment suppressed senescence by inhibiting miR-34a-3p.

### 3.7. CAB39 Alleviates miR-34a-3p-Induced Senescence in DPSCs through the AMPK/mTOR Signaling Pathway

**Figure 7:**
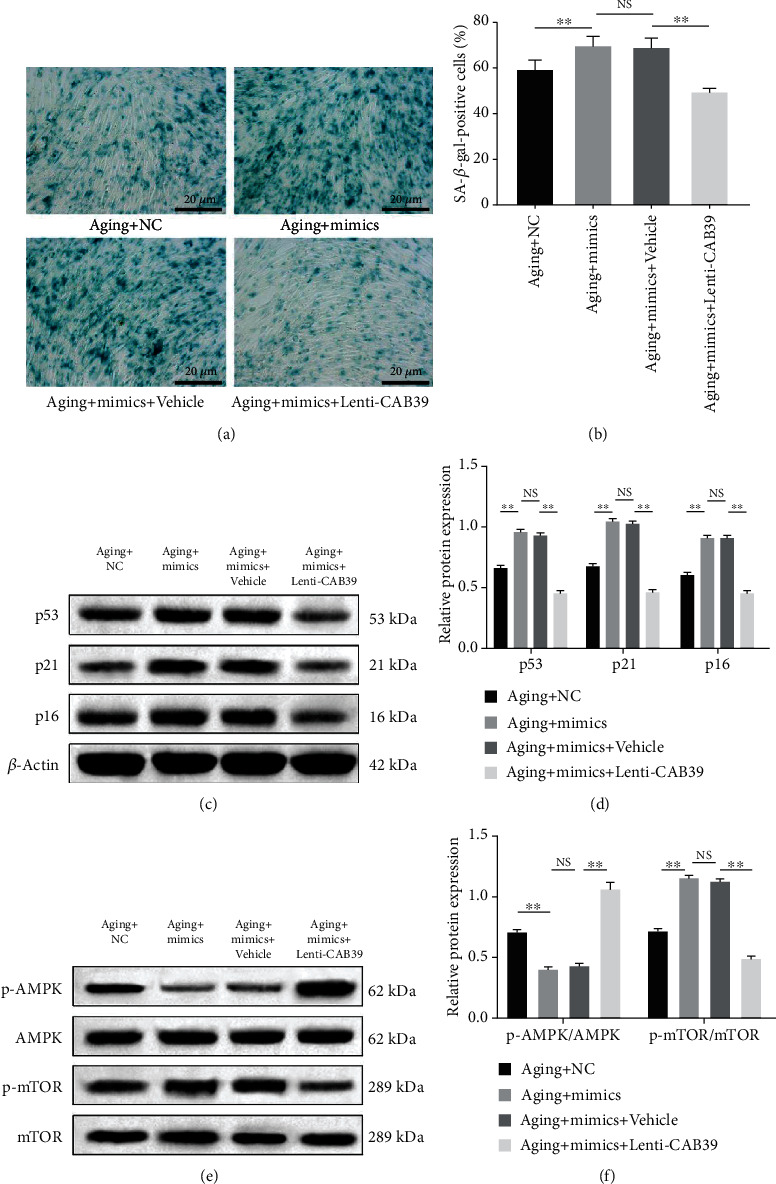
CAB39 alleviates miR-34a-3p-induced senescence in DPSCs through the AMPK/mTOR signaling pathway. Aging DPSCs were pretransfected with miR-34a-3p mimics or miR-NC for 5 h, prior to transfection with Lenti-CAB39 or vehicle control for 48 h. (a) Representative images of SA-*β*-gal staining and (b) quantitative analysis of SA-*β*-gal-positive cells (scale bar: 20 *μ*m). (c) Representative blots and (d) quantitative analysis of the expression levels of p53, p21, and p16. (e) Representative blots of the expression levels of p-AMPK, AMPK, p-mTOR, and mTOR. (f) Quantitative analysis of p-AMPK/AMPK and p-mTOR/mTOR ratios. Data are expressed as mean ± standard deviation (*n* = 3). NS: not significantly different. ^∗∗^*p* < 0.01, with comparisons indicated by lines.

To explore the effect of CAB39 on miR-34a-3p-induced DPSC senescence, CAB39 lacking the miR-34a-3p target site was overexpressed in aging DPSCs, and the cells were treated with miR-34a-3p mimics. After transfection with Lenti-CAB39, the miR-34a-3p-induced increase in the positive rate of SA-*β*-gal staining was significantly decreased (*p* < 0.01), whereas no statistical difference was observed in the vehicle-treated control group (*p* > 0.05, Figures 7(a) and 7(b)). Furthermore, western blotting results established that Lenti-CAB39 treatment significantly reversed the alteration of several critical senescence-associated proteins induced by miR-34a-3p mimics, including an increase in the expression levels of p53, p21, p16, and p-mTOR and a reduction in the expression of p-AMPK (*p* < 0.01, Figures 7(c)–7(f)). Overall, these results suggested that CAB39 could rescue miR-34a-3p-induced senescence in DPSCs by modulating the AMPK/mTOR signaling pathway.

## 4. Discussion

Cellular senescence is an inevitable biological phenomenon that is associated with age or with prolonged culture *in vitro*. It is characterized by a general degeneration in physiological functions. Stem cell senescence is considered a major cause of the declines in tissue and organ functions and is likely to affect the efficacy of stem cell-based therapies [30]. In this study, metformin treatment was, for the first time, shown to upregulate CAB39 and activate the AMPK/mTOR signaling pathway by downregulating miR-34a-3p, which alleviated DPSC senescence. This study elucidates on the inhibitory effect of metformin on DPSC senescence and provides a basis for the potential clinical application of metformin in delaying senescence.

In this study, human DPSCs were successfully isolated from the dental pulp tissues of freshly extracted teeth. The cultured DPSCs displayed a fibroblast-like spindle-shaped morphology. Both young and aging DPSCs positively expressed typical mesenchymal surface markers (CD29, CD90, and CD105), while barely expressing hematopoietic markers (CD11b, CD34, CD45, and CD14). The expression of cell surface antigens was consistent with the criteria for MSCs [31]. These results suggested that the isolated young and aging DPSCs were identified as MSCs.

Previous studies have reported that metformin mediated cell proliferation in a dose-dependent manner; therefore, determination of a suitable drug dosage for use in *in vitro* studies is extremely important to obtain the desired effect [32, 33]. It has been reported that 100 and 200 *μ*M metformin exerted the greatest proproliferative effect on osteoblast-like cells [34]. Similarly, another study found that 100 *μ*M metformin enhanced the proliferative activity of DPSCs [35]. In the present study, 100 *μ*M metformin was found to have the best enhancing effect on the proliferation of DPSCs. However, the results demonstrated that a high concentration of 500 *μ*M metformin exerted antiproliferative effects towards DPSCs, thus limiting its application.

The inhibitory role of metformin in various senescence processes has been elucidated in multiple model organisms and human cell lines. Metformin has been found to have a gero-therapeutic effect on stem cells, including targeting stem cell exhaustion, delaying cellular attrition, maintaining cellular function, and preventing premature senescence. As previously reported, metformin inhibited the senescence of intestinal stem cells (ISCs) in *Drosophila* and enhanced the lifespan of human MSCs [15, 36]. Recently, metformin was found to partially reverse the dysregulation of rejuvenation and differentiation ability of aged oligodendrocyte progenitor cells (OPCs), further restoring the remyelination capacity [37]. This study focused on the effects of metformin on young and aging DPSCs, and the results confirmed the gero-suppressive effect as evidenced by a significant decrease in SA-*β*-gal activity and the expression levels of senescence-associated proteins.

AMPK functions as a key nutrient and energy sensor, which is involved in diverse pathophysiological processes [38]. Studies have reported that metformin indirectly induces AMPK to prevent cellular senescence caused by a decrease in AMPK activity in aging MSCs [39]. Besides, AMPK is considered to be a key factor involved in blocking mTOR signaling, an essential regulator of senescence. Activation of mTOR leads to a reduction in autophagy, thereby promoting cellular senescence [40]. Accumulating evidence indicates that the AMPK/mTOR signaling pathway is involved in the control of senescence [41], and metformin exhibits an inhibitory role in the phosphorylation of mTOR by activating AMPK [19]. Furthermore, CAB39, a component of the trimeric LKB1-STRAD-CAB39 complex, contributes to the stabilization of LKB1 to STRAD binding and is recognized as an upstream activator of AMPK due to its role in activating AMPK signaling by phosphorylating AMPK*α*1 on residue Thr-172 [42]. It has been reported that CAB39 is associated with MSC senescence, which occurs in an AMPK-dependent manner [43]. In the current study, our results showed a decrease in the expression of CAB39 and p-AMPK and an increase in the expression of p-mTOR in aging DPSCs when compared to young DPSCs. Remarkably, following metformin treatment, the expression levels of CAB39 and p-AMPK were upregulated in both young and aging DPSCs, while p-mTOR was downregulated. Taken together, these results imply that the signaling activity of CAB39/AMPK/mTOR is associated with a metformin-mediated gero-suppressive effect.

In recent years, miR-34a, a member of the conserved miR-34 family, has been intensively studied. Previous studies document that miR-34a is involved in multiple biological processes, including cell cycle, development, differentiation, apoptosis, and senescence [44–46]. Inhibition of miR-34a reduces senescence in human adipose-derived mesenchymal stem cells (AMSCs) [46]. In a previous study, microarray analysis revealed that miR-34a is significantly elevated in aging DPSCs, indicating that it could be a vital age-related miRNA of DPSCs [24]. Our results are consistent with previous findings that miR-34a-3p was highly expressed in aging DPSCs. Additionally, metformin has been shown to play an important role in the regulation of the miR-34a expression [25–28]. In this study, metformin exhibited inhibitory effects on the expression of miR-34a-3p. Further studies showed that overexpression of miR-34a-3p promoted cellular senescence in aging DPSCs, while metformin significantly attenuated miR-34a-3p-mediated senescence through the activation of the AMPK/mTOR signaling pathway. Moreover, miR-34a-3p downstream targets were identified, and CAB39 was found to be a direct target gene of miR-34a-3p. Treatment with miR-34a-3p mimics significantly reduced the expression of CAB39 and attenuated the metformin-induced upregulation of CAB39. Notably, enhancement of senescence in DPSCs by miR-34a-3p mimics was partially reversed by Lenti-CAB39 transfection. In addition, involvement of the AMPK/mTOR signaling pathway was also confirmed in this process. Collectively, we established that metformin-induced miR-34a-3p downregulation alleviates DPSC senescence by targeting CAB39 through the AMPK/mTOR signaling pathway. However, AMPK/mTOR possesses various bypass signaling cascades that modulate its functions; besides CAB39, we cannot rule out that miR-34a-3p regulates the activation of the AMPK/mTOR signaling pathway through other molecules or pathways.

## 5. Conclusions

In summary, we found that metformin inhibits DPSC senescence by downregulating miR-34a-3p, which leads to the upregulation of CAB39 and the activation of the AMPK/mTOR signaling pathway. These results indicate that metformin can be used to alleviate cellular senescence in DPSCs, and the miR-34a-3p-CAB39/AMPK/mTOR axis may be a novel therapeutic target of metformin which alleviates senescence.

## Data Availability

The data used to support the findings of this study are available from the corresponding author upon request.
